# Supplementary material for: EnzML: multi-label prediction of enzyme classes using InterPro signatures
Source: BMC Bioinformatics. 2012 Apr 25;13:61. doi: 10.1186/1471-2105-13-61 (PMC3483700; doi:10.1186/1471-2105-13-61)
Supplement: Addtional file 5 — The Java code to format the data files, evaluate and predict. The file enzml_java_code.tar.gz contains the Java code used to format database data to ARFF and XML formats, to execute cross and train-test (jackknife) evaluations and to record evaluation results to database. More information is included in the readme.txt file and the Javadoc files. The code can be used with a MySQL database. To use a different database software, other JDBC drivers might be required. [file 1471-2105-13-61-S5.gz › java_code/utils/doc/uk/ac/ed/inf/utils/diff/Diff.html]

Diff


---


|  |  |  |
| --- | --- | --- |
| **Overview** \| **Package** \| **Class** \| **Use** \| **Tree** \| **Deprecated** \| **Index** \| **Help** | |  |
| PREV CLASS   **NEXT CLASS** | **FRAMES**    **NO FRAMES**     **All Classes** |  |
| SUMMARY: NESTED \| FIELD \| CONSTR \| METHOD | DETAIL: FIELD \| CONSTR \| METHOD |  |


---


## uk.ac.ed.inf.utils.diff Class Diff

```
java.lang.Object
  uk.ac.ed.inf.utils.diff.Diff
```

---

``` public class Diff extends java.lang.Object ```

Compares two collections, returning a list of the additions, changes, and
deletions between them. A `Comparator` may be passed as an
argument to the constructor, and will thus be used. If not provided, the
initial value in the `a` ("from") collection will be looked at to
see if it supports the `Comparable` interface. If so, its
`equals` and `compareTo` methods will be invoked on the
instances in the "from" and "to" collections; otherwise, for speed, hash
codes from the objects will be used instead for comparison.

The file FileDiff.java shows an example usage of this class, in an
application similar to the Unix "diff" program.

---

**Constructor Summary**

`Diff(java.util.Collection a,
java.util.Collection b)`
  
          Constructs the Diff object for the two collections, using the default
comparison mechanism between the objects, such as `equals` and
`compareTo`.

`Diff(java.util.Collection a,
java.util.Collection b,
java.util.Comparator comp)`
  
          Constructs the Diff object for the two collections, using the given
comparator.

`Diff(java.lang.Object[] a,
java.lang.Object[] b)`
  
          Constructs the Diff object for the two arrays, using the default
comparison mechanism between the objects, such as `equals` and
`compareTo`.

`Diff(java.lang.Object[] a,
java.lang.Object[] b,
java.util.Comparator comp)`
  
          Constructs the Diff object for the two arrays, using the given
comparator.


| **Method Summary** | |
| --- | --- |
| `java.util.List` | `diff()`             Runs diff and returns the results. |
| `java.lang.Integer[]` | `getLongestCommonSubsequences()`             Returns an array of the longest common subsequences. |

**Methods inherited from class java.lang.Object**

`equals, getClass, hashCode, notify, notifyAll, toString, wait, wait, wait`

**Constructor Detail**

### Diff

```
public Diff(java.util.Collection a,
            java.util.Collection b)
```

:   Constructs the Diff object for the two collections, using the default
    comparison mechanism between the objects, such as `equals` and
    `compareTo`.

---


### Diff

```
public Diff(java.util.Collection a,
            java.util.Collection b,
            java.util.Comparator comp)
```

:   Constructs the Diff object for the two collections, using the given
    comparator.

---


### Diff

```
public Diff(java.lang.Object[] a,
            java.lang.Object[] b)
```

:   Constructs the Diff object for the two arrays, using the default
    comparison mechanism between the objects, such as `equals` and
    `compareTo`.

---


### Diff

```
public Diff(java.lang.Object[] a,
            java.lang.Object[] b,
            java.util.Comparator comp)
```

:   Constructs the Diff object for the two arrays, using the given
    comparator.


**Method Detail**

### diff

```
public java.util.List diff()
```

:   Runs diff and returns the results.

---


### getLongestCommonSubsequences

```
public java.lang.Integer[] getLongestCommonSubsequences()
```

:   Returns an array of the longest common subsequences.


---


|  |  |  |
| --- | --- | --- |
| **Overview** \| **Package** \| **Class** \| **Use** \| **Tree** \| **Deprecated** \| **Index** \| **Help** | |  |
| PREV CLASS   **NEXT CLASS** | **FRAMES**    **NO FRAMES**     **All Classes** |  |
| SUMMARY: NESTED \| FIELD \| CONSTR \| METHOD | DETAIL: FIELD \| CONSTR \| METHOD |  |


---
